# Hydraulic conductivity of human cancer tissue: A hybrid study

**DOI:** 10.1002/btm2.10617

**Published:** 2023-11-23

**Authors:** Hooman Salavati, Pim Pullens, Charlotte Debbaut, Wim Ceelen

**Affiliations:** ^1^ Department of Human Structure and Repair Ghent University Ghent Belgium; ^2^ IBiTech–BioMMedA, Ghent University Ghent Belgium; ^3^ Cancer Research Institute Ghent (CRIG) Ghent Belgium; ^4^ Department of Radiology University Hospital Ghent Ghent Belgium; ^5^ Ghent Institute of Functional and Metabolic Imaging (GIFMI) Ghent University Ghent Belgium; ^6^ IBiTech–Medisip, Ghent University Ghent Belgium

**Keywords:** chemical engineering‐based bioprocessing, computational modeling, drug delivery, tissue engineering

## Abstract

**Background:**

Elevated tumor tissue interstitial fluid pressure (IFP) is an adverse biomechanical biomarker that predicts poor therapy response and an aggressive phenotype. Advances in functional imaging have opened the prospect of measuring IFP non‐invasively. Image‐based estimation of the IFP requires knowledge of the tissue hydraulic conductivity (*K*), a measure for the ease of bulk flow through the interstitium. However, data on the magnitude of *K* in human cancer tissue are not available.

**Methods:**

We measured the hydraulic conductivity of tumor tissue using modified Ussing chambers in surgical resection specimens. The effect of the tumor microenvironment (TME) on *K* was investigated by quantifying the collagen content, cell density, and fibroblast density of the tested samples using quantitative immune histochemistry. Also, we developed a computational fluid dynamics (CFD) model to evaluate the role of *K* on interstitial fluid flow and drug transport in solid tumors.

**Results:**

The results show that the hydraulic conductivity of human tumor tissues is very limited, ranging from approximately 10^−15^ to 10^−14^ m^2^/Pa∙s. Moreover, *K* values varied significantly between tumor types and between different samples from the same tumor. A significant inverse correlation was found between collagen fiber density and hydraulic conductivity values. However, no correlation was detected between *K* and cancer cell or fibroblast densities. The computational model demonstrated the impact of K on the interstitial fluid flow and the drug concentration profile: higher *K* values led to a lower IFP and deeper drug penetration.

**Conclusions:**

Human tumor tissue is characterized by a very limited hydraulic conductivity, representing a barrier to effective drug transport. The results of this study can inform the development of realistic computational models, facilitate non‐invasive IFP estimation, and contribute to stromal targeting anticancer therapies.

**Translational Impact Statement**

The effectiveness of drug delivery to solid tumors is hindered by elevated interstitial fluid pressure (IFP), which is strongly associated with the hydraulic conductivity (*K*) of tumor stroma. However, accurate *K* values in human cancer tissue are lacking. We introduce a novel method for measuring *K* in clinical samples. Our results reveal substantial inter‐ and intra‐tumor variations in *K* values, impacting IFP and drug delivery. The findings can provide valuable insights for developing innovative therapeutic strategies that specifically target the biomechanical tumor environment.

## 1 INTRODUCTION

Cancer is a major cause of death worldwide.^1^ The efficacy of systemic cancer therapies is limited by poor drug penetration in tumor tissue.^2^ The major obstacle for tissue penetration of anticancer drugs is the elevated interstitial fluid pressure (IFP), an adverse biomarker of treatment response and prognosis.^2^, ^3^, ^4^, ^5^, ^6^, ^7^, ^8^ In normal tissues, the IFP is generally around the atmospheric pressure (reference value of 0 Pa).^9^, ^10^ In contrast, IFP values as high as 5.3 kPa (40 mmHg) and higher were recorded in a variety of solid tumors including head and neck, breast, and cervix cancer.^4^, ^11^ Elevated tumor IFP is caused by structural and functional alterations including hyperpermeable microvessels, deficient lymphatic drainage, and excessive deposition of interstitial structural fibers.^4^, ^12^, ^13^

Until recently, measurement of IFP as a biomarker in clinical oncology was limited to invasive methods requiring insertion of a probe or needle.^2^ However, advances in functional imaging such as dynamic contrast enhanced MRI have opened the prospect of non‐invasive estimation of tumor IFP.^14^, ^15^, ^16^, ^17^, ^18^, ^19^, ^20^, ^21^, ^22^, ^23^ Image‐based estimation of the IFP is possible by observing, during dynamic image acquisition, the resulting (outward) convective flow of interstitial fluid. Assuming solid tumors to be a porous medium, the relation between interstitial fluid velocity (IFV) and the IFP gradient is described by Darcy's law^24^:

(1) $$u_{i}=-K\nabla p_{i}$$

with $u_{i}$ [m/s] the IFV, K [m^2^/Pa·s] the hydraulic conductivity of the tumor tissue, and $p_{i}$ [Pa] the IFP. The hydraulic conductivity (*K*), which represents the ease of bulk flow through the interstitium, is correlated with the IFP.^24^ Estimation of the IFP using image‐based methods therefore requires knowledge of *K*. The hydraulic conductivity of tumor tissue is known to be spatially heterogenous, and is determined by the permeability of extracellular matrix (ECM) as well as the properties of interstitial fluid.^25^, ^26^ Hydraulic conductivity can be written as the ratio of tissue permeability (*k* [m^2^]) and interstitial fluid dynamic viscosity (*μ* [Pa·s])^24^:

(2) $$K=\frac{k}{\mu }$$

Whereas measurements of interstitial fluid viscosity are rather straightforward,^27^ the estimation of *k* is more challenging.^28^ According to *the* Kozeny–Carman (KC) equation,^29^ k can be estimated as a function of tissue porosity (*ε* [−]) and the ratio of the total surface area of the pore boundaries to the total bulk volume (*α* [1/m]):

(3) $$k=\frac{\varepsilon^{3}}{Q\alpha^{2}}$$

*Q* [−] is the porosity‐dependent KC constant which for *ε* < 0.7 is approximately equal to 5.^30^ Regarding the limited knowledge on the dependency of *α* on *ε*, the relationship between *k* and *ε* in solid tumors is complex.^30^ However, some studies have suggested positive correlations between tumor tissue *k* and *ε*,^31^, ^32^, ^33^ indicating that an increase in porosity leads to an increase in permeability.

The diffusion of anticancer agents is also influenced by the architecture of the tumor tissue, particularly the *ε* and tortuosity (*τ*) of the extracellular matrix (ECM).^34^ Within solid tumors, the effective diffusion coefficient (*D*_eff_) is generally smaller than the diffusion coefficient in free fluid (*D*) due to the constraints imposed by the solid components of the ECM.^35^, ^36^ This phenomenon can be expressed mathematically as^37^:

(4) $$D_{\mathrm{eff}}=\mathrm{\varepsilon D}$$

Other approximations of *D*_eff_ based on *ε* were also introduced with a similar trend (see, for instance^38^), suggesting a direct relation between *D*_eff_ and *K*, as higher *ε* can lead to higher *K*.

Preclinical studies have attempted to measure *K* in cancer tissue. Swabb et al.^39^ were the first to publish *K* values of cancer (Morris hepatoma 5123) as well as normal (subcutaneous) tissue, and showed higher values of *K* in tumor (e.g., 3.1 *×* 10^−14^ m^2^/Pa·s) compared to normal tissue (e.g., 6.4 *×* 10^−15^ m^2^/Pa·s). The same group developed in vitro and in vivo methods to monitor the fluid exchange through samples obtained from female Buffalo rats.^40^ The authors found a strong inverse correlation between *K* and the glycosaminoglycan (GAG) content of the samples.

Boucher et al.^41^ used Darcy's law to estimate *K* in a mouse colorectal cancer model by generating an infusive flow and measuring the IFP using the micropipette method simultaneously; measured values were around 1.5 *×* 10^−13^ m^2^/Pa·s. Using the same animal model and implementing a modified in‐vitro method described by Swabb et al.,^33^ Znati et al.^42^ measured *K* (in the order of 10^−13^ m^2^/Pa·s) and established an inverse association with collagen fiber density. The authors also found that a radiotherapy induced increase of collagen fiber production was a major cause for reducing *K*.

Apart from studies using realistic human tumor and vascular geometries,^43^, ^44^ so far, simulations and studies of interstitial physiology and drug delivery had to rely on animal data for biophysical properties.^7^, ^22^, ^45^, ^46^, ^47^, ^48^, ^49^, ^50^, ^51^, ^52^ Here, we present the first comprehensive study of *K* measured in human tumor tissue samples. Also, we have studied the correlation of hydraulic conductivity in these samples with the structure of the ECM, and developed a computational fluid dynamics (CFD) model to evaluate the role of *K* on interstitial fluid flow and drug transport in solid tumors.

## 2 METHODS

### 2.1 Measurement of hydraulic conductivity

The study was approved by the institutional review board of Ghent University Hospital (EC/2017/0784), and all patients provided written informed consent. Fresh samples (*n* = 20) were harvested from 18 patients with peritoneal metastases or pancreatic cancer (Table 1).

**TABLE 1:** Overview of included tumor types.

Tissue type	Cancer type	Tissue location	No. of patients
Primary tumor	Pancreatic (PC)	‐	3
Peritoneal metastasis (PM)	Colorectal (CRC)	Peritoneum	5
Peritoneal metastasis (PM)	Colorectal (CRC)	Abdominal wall (AW)	3
Peritoneal metastasis (PM)	Small bowel (SB)	Peritoneum	1
Peritoneal metastasis (PM)	Ovarian (OC)	Peritoneum	5
Ovarian metastasis (OM)	Small bowel (SB)	Ovary	1
Normal peritoneum (NP)	‐	Peritoneum	2

**FIGURE 1:**
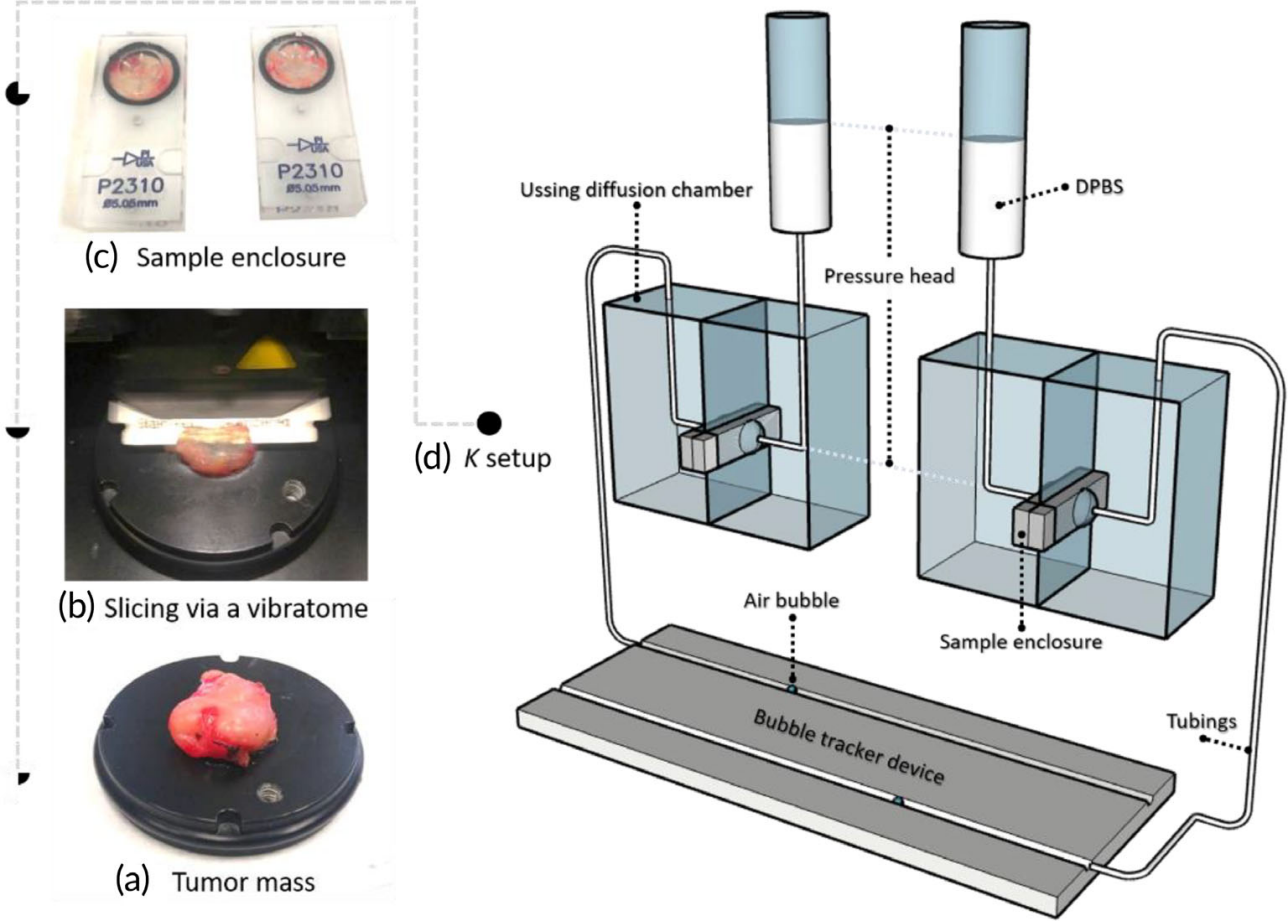
Ex‐vivo setup for measuring *K* in human cancer tissue. (a–c) After harvesting samples, they were sliced (1 mm thickness), punched (with a diameter of 12 mm), and placed on sample enclosures of the Ussing diffusion chamber. (d) The *K* measurement mainly includes two modified Ussing diffusion chambers, two DPBS reservoirs for generating the hydrostatic pressure, and a bubble tracker device for quantifying the buffer exchange through the samples.

Samples were glued on a plastic plate (Figure 1a) and multiple 1 mm thick slices were cut with a vibrating Microtome (VT1200S, Leica Biosystems, Nussloch, Germany; Figure 1b). The slices were shaped into circular discs with a diameter of 12 mm using a flat‐ended cylindrical punch. Afterwards, the samples were placed in the enclosures of two Ussing diffusion chambers (Figure 1c). The hydraulic conductivity of the samples was measured by detecting the fluid exchange through the sample in a closed system due to a hydrostatic pressure (with a pressure head of 16 cm H_2_O), which corresponds to a realistic value of 1.6 kPa (11.8 mmHg) mimicking the elevated IFP (Figure 1d). The system included two modified Ussing diffusion chambers placed in a 2‐channel Easy Mount stand (Physiologic Instruments Inc., San Diego, USA), pressure reservoirs (syringe reservoirs), and a bubble tracker device for measuring the liquid exchange.

The design of the apparatus was adopted from the work of Helton et al.,^53^ who used it to measure the hydraulic conductivity of the human blood‐nerve barrier. The hydrostatic pressure was generated by a column of Dulbecco's phosphate buffered saline 1X (DPBS 1X; Thermo Fisher Scientific, Waltham, MA, USA). The bubble tracker device included a custom made breadboard used to assemble a slotted plate for holding two plastic tubes (1.00 mm internal diameter; Ace Glass, Vineland, NJ). In each Ussing chamber, the openings were sealed, except for one of the top openings of each side. The syringe reservoirs were connected to the feeding sides of the diffusion chambers with precision tubing. The outlet sides of the diffusion chambers were linked to the bubble tracker with precision tubing (Figure 1d). Prior to the experiments, the tubes were filled with PBS buffer, and an air bubble was loaded by injection into each tube of the bubble. After testing the setup for leakage, the static pressure was maintained by adding PBS in each reservoir, and the fluid flux was measured by imaging the location of the bubbles every 10 min during 2 h.

The equation to calculate the nominal hydraulic conductivity (*K′*) was derived from Darcy's law (Equation (1)):

(5) $$K^{\prime }={\left(\frac{b}{d}\right)}^{2}\frac{w}{\mathrm{\rho gh}}\frac{dx}{dt}$$

where *w* [m] and *d* [m] are the tissue thickness and tissue wet diameter (calculated based on the area exposed to the fluid), *b* [m] the tube diameter, *ρ* [kg/m^3^] the liquid density, *h* [m] the liquid column height (determined by subtracting the pressure heads at the feeding and outlet sides), *g* [m/s^2^] the gravity acceleration, and $\frac{dx}{dt}$ [m/s] the velocity of the air bubble displacement (Table 2).

**TABLE 2:** Parameter values of ex‐vivo testing.

Parameter	Value range	Reference
w $\left[m\right]$	$1\times 10^{-3}$	‐
d $\left[m\right]$	$5.05\times 10^{-3}$	‐
b $\left[m\right]$	$1\times 10^{-3}$	‐
ρ [kg/m^3^]	998	56
h $\left[m\right]$	$1.6\times 10^{-1}$	‐
$\mu_{\mathrm{PBS}}$ $\left[\mathrm{Pa}.s\right]$	$8.9\times 10^{-4}\;\left[25{}^{\circ}C\right]$ to 1$.1\times 10^{-3}\;\left[18{}^{\circ}C\right]$	55
$\mu_{\text{IF}}$ $\left[\mathrm{Pa}.s\right]$	$3.5\times 10^{-3}$	27

The tests were done at room temperature, and the operating temperature for each test was recorded (ranging between 18 and 25°C). Assuming that the viscosity of PBS buffer (*μ*_PBS_) is similar to that of water,^54^ the value of *μ*_PBS_ was estimated with respect to the room temperature measurements (i.e., from^55^). Afterwards, the values of *K′* were corrected to obtain estimated *K* values using the viscosity of interstitial fluid viscosity (*μ*_IF_) at body temperature (see also Table 2):

(6) $$K=\frac{\mu_{\mathrm{PBS}}}{\mu_{\text{IF}}}K^{\prime }$$

### 2.2 Quantitative histology

To evaluate how *K* is related with the structure of the TME, we estimated collagen content, cell density, and fibroblast density of the samples using immune histochemistry combined with image analysis. After measurement of *K* the samples were paraffin embedded, and multiple slices with a thickness of 3 μm were cut with a microtome. Samples were stained with hematoxylin and eosin (H&E), Masson's trichrome and alpha‐smooth muscle actin (ASMA) to investigate the cell density, fiber density and fibroblast density, respectively (detailed methods are provided in Appendix S1).

**FIGURE 2:**
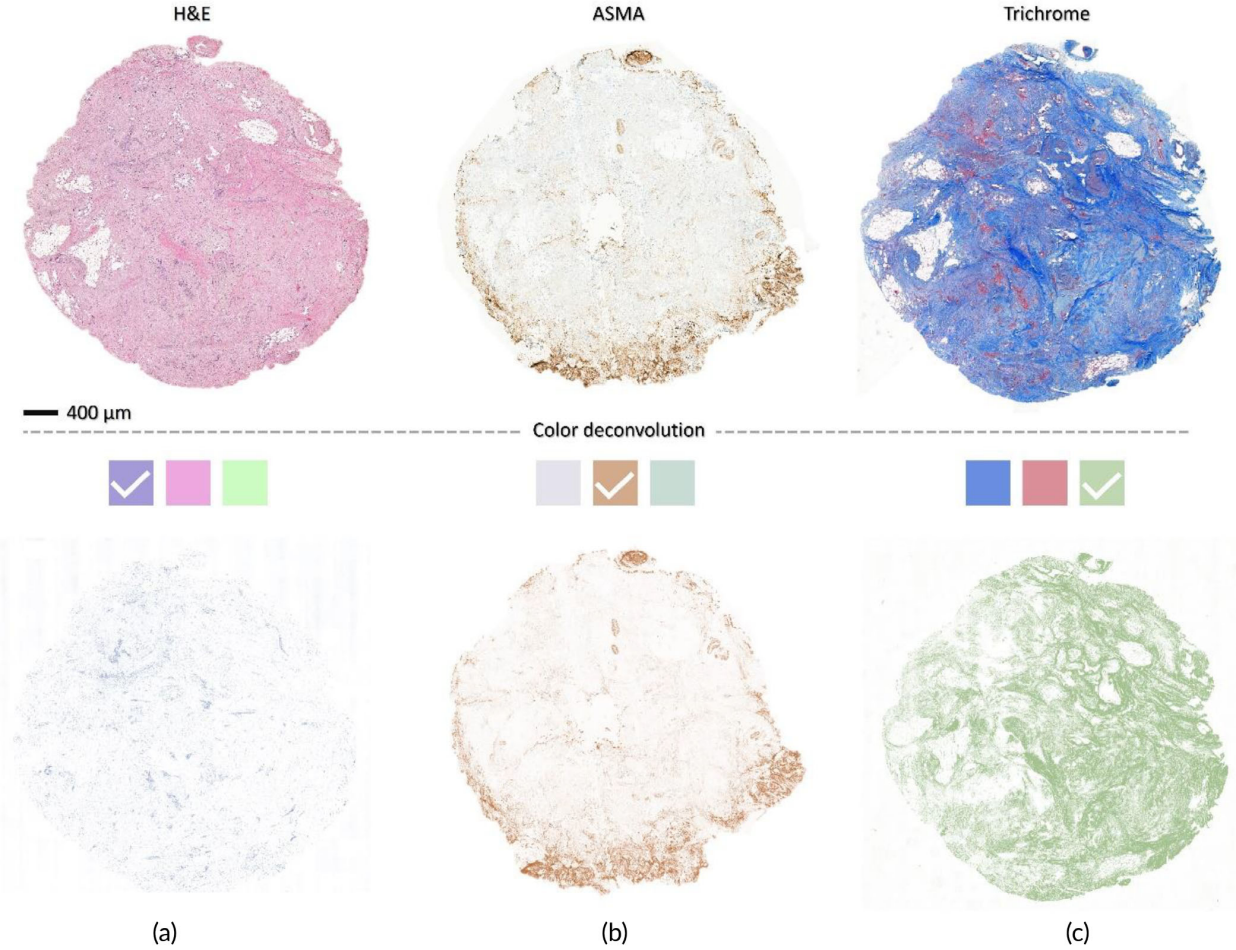
The TME elements quantification procedures. After the color deconvolution procedure for each stain, the proper channels were selected to quantify the area (%) covered by the (a) cells, (b) fibroblasts, and (c) collagen fibers.

The stained slides were scanned digitally with a Panoramic 250 Flash III scanner (3DHISTECH, Budapest, Hungary) and imported into the open‐source software QuPath^57^ for manually determining the region of interest (ROI). Subsequently, the selected ROIs were sent to ImageJ software for quantifying the fiber, fibroblast and cell densities using color deconvolution techniques (Figure 2).^58^, ^59^, ^60^

### 2.3 CFD modeling

**FIGURE 3:**
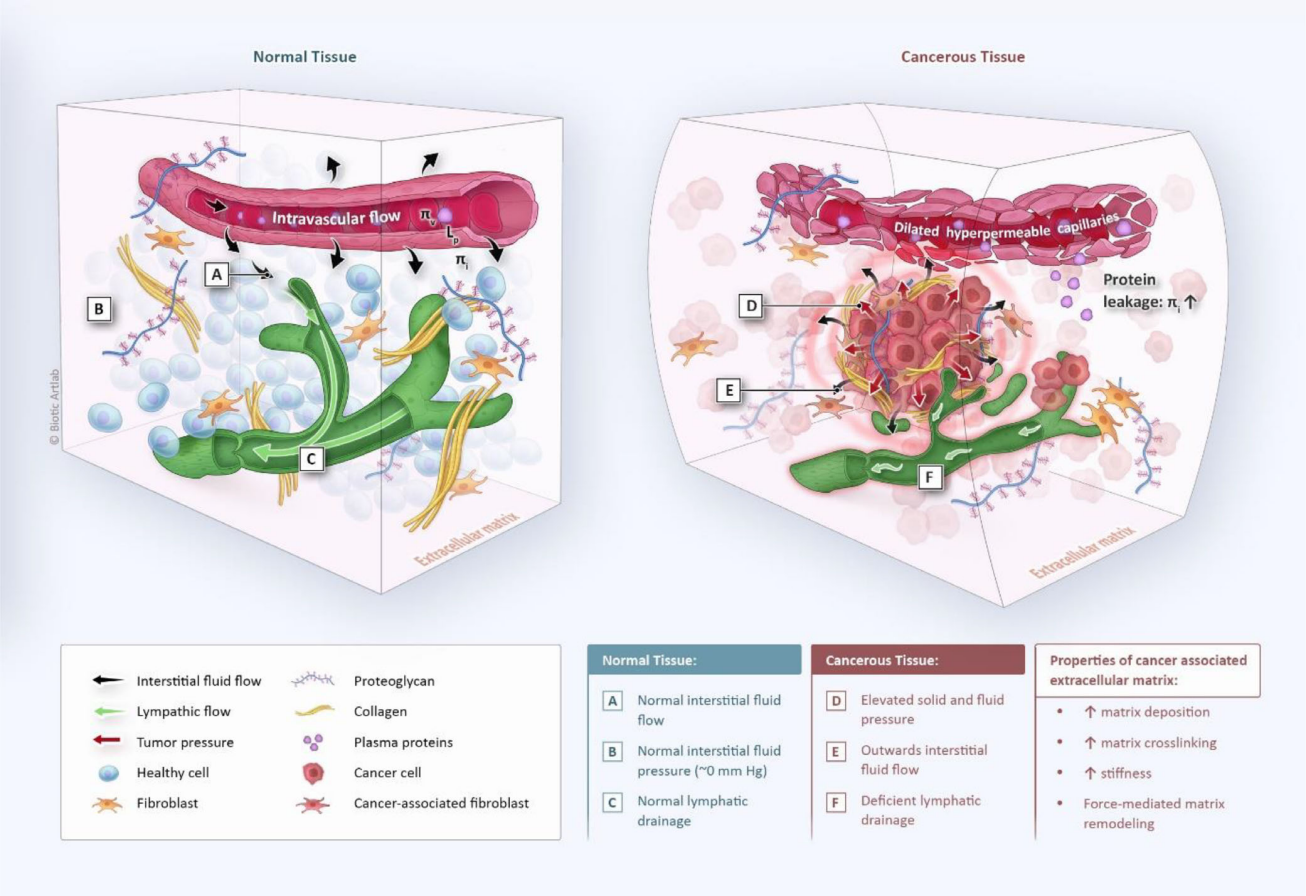
Normal and cancerous tissues distinct tissue architecture and biophysical properties. In normal tissue (left), fluid equilibrium is maintained by a balance between fluid inflow (i.e., *J*_V_) and outflow (i.e., *J*_L_), resulting in a low (∼0 Pa) interstitial fluid pressure (IFP). Conversely, cancerous tissue (right) exhibits structural and functional deficiencies in blood and lymphatic microvessels, accompanied by heightened matrix deposition and cancer cell proliferation. Consequently, these alterations lead to augmented solid and fluid pressure and increased matrix stiffness, causing interstitial fluid to leak radially outward.

A computational fluid dynamics (CFD) model was created to investigate the role of *K* in interstitial fluid flow and drug transport inside solid tumors. The transport of interstitial fluid through normal tissue interstitium is determined by the balance between inward flow from the blood capillaries and drainage by the lymphatic network.^2^ In tumor tissue, an imbalance is generated due to the abnormal leakiness of the tumor capillary network and the (partial) absence of functional lymphatic drainage (Figure 3). Assuming tumor interstitium to be an isotropic porous medium, the interstitial flow regimen is described by (i) Darcy's law (Equation (1))^7^ and (ii) the continuity equation (Equation (7)), where the rate of inward flow (*J*_V_ [s^−1^]) is described by Starling's law (Equation (8)) and lymphatic drainage (*J*_L_ [s^−1^]) in normal tissue is described by Equation (9)^24^:

(7) $$\nabla ∙u_{i}=J_{V}-J_{L}$$

(8) $$J_{V}=L_{p}\frac{S}{V}\left(p_{v}-p_{i}-\sigma \left(\pi_{v}-\pi_{i}\right)\right)$$

(9) $$J_{L}=L_{p_{L}}\frac{S_{L}}{V_{L}}\left(p_{i}-p_{L}\right)$$

where $L_{p}$ [m/Pa/s] represents the hydraulic conductivity of the vasculature, S/V [1/m] the surface area of blood vessels per unit volume for transport in the tumor interstitium, and $p_{v}$ [Pa], $p_{i}$ [Pa], σ [−], $\pi_{v}$ [Pa] and $\pi_{i}$ [Pa] the vascular pressure, interstitial fluid pressure, osmotic reflection coefficient, vascular and interstitial osmotic pressure, respectively. In the surrounding normal tissue, $L_{p_{L}}$ [m/Pa·s] represents the hydraulic conductivity of lymphatic vessels, $S_{L}/V_{L}$ [1/m] is the surface area of lymphatic vessels per unit volume, and $p_{L}$ [Pa] is the effective lymphatic pressure.

Taking into account both vascular ($\varphi_{V}$) and lymphatic ($\varphi_{L}$) uptake of the drug in the tissue, drug transport can be modeled as^7^:

(10) $$\frac{dC}{dt}=D_{\mathrm{eff}}\nabla^{2}C-\nabla \cdot \left(u_{i}C\right)+\varphi_{V}-\varphi_{L}$$

where C [mol/m^3^] and $D_{\mathrm{eff}}$ [m^2^/s] are the interstitial drug concentration and the drug's effective diffusion coefficient, respectively. Mathematically, to our knowledge, no published values of $D_{\mathrm{eff}}$ based on *K* are available for tumor and healthy tissue. However, by analyzing the published data of a mathematical framework modeling local drug delivery^32^ and an in vitro study investigating breast tumor tissue permeability,^31^ we described *K* as a power function of *ε* (i.e., *K* ∝ *ε* ^*3*^), *subsequently* enabling the definition of $D_{\mathrm{eff}}$ based on *K*:

(11) $$\frac{D_{\mathrm{eff}}}{D_{\mathrm{eff}}^{{}^{\circ}}}=\sqrt[{3}]{\frac{K}{K^{{}^{\circ}}}}$$

where $D_{\mathrm{eff}}^{{}^{\circ}}$ and $K^{{}^{\circ}}$ are the baseline values for the effective diffusion coefficient and hydraulic conductivity, respectively. $\varphi_{V}$ and $\varphi_{L}$ are then described by the following equations^7^, ^45^:

(12) $$\varphi_{V}=L_{d}\frac{S}{V}\left(c_{p}-C\right)\left(\frac{\mathrm{Pe}}{e^{\mathrm{Pe}}-1}\right)+J_{V}\left(1-\sigma_{d}\right)c_{p}$$

(13) $$\varphi_{L}=J_{L}C$$

where $L_{d}$ [m/s] is the vascular permeability for the drug coefficient, $c_{p}$ [mol/m^3^] is the plasma concentration of the drug, and $\sigma_{d}$ [−] is the solvent‐drag reflection coefficient describing the ease of drug transport across the tumor vasculature.^7^ It was assumed that $c_{p}$ remains negligible (i.e., $c_{p}$ = 0) during drug administration based on a relatively short therapeutic period (typically 30–60 min).^45^ Pe [−], the Peclet number, represents the ratio of convective to diffusive transport^24^:

(14) Pe=Jv1−σdLdSV

The values of *K* were obtained from the ex vivo measurements in this study, and the values of the parameters in Equations ((8), (9), (10), (11), (12), (13), (14)) are listed in Table S1.

**FIGURE 4:**
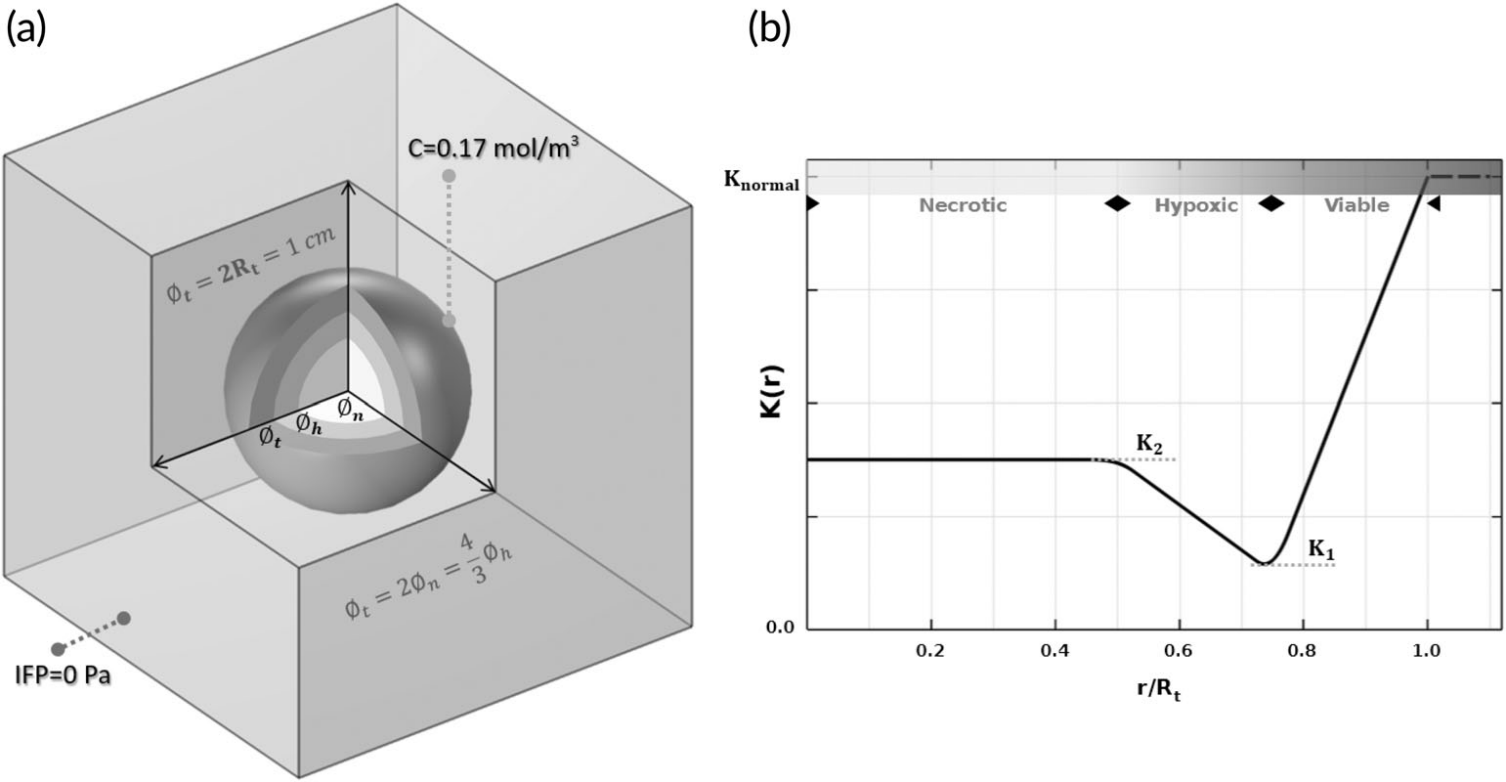
(a) Computational domain comprising a spherical solid tumor (with a total diameter of Ø_t_ = 2*R*_t_ = 10 mm) and the surrounding normal tissue. The tumor consists of a necrotic core (with a diameter of Ø_n_ = 5 mm), a hypoxic region (with a thickness of 2.5 mm) and the rest is viable region. (b) the prescribed profile for intra‐tumoral heterogeneity of *K*. The values of *K*_1_, *K*_2_, and *K*_normal_ were based on the ex vivo results.

**FIGURE 5:**
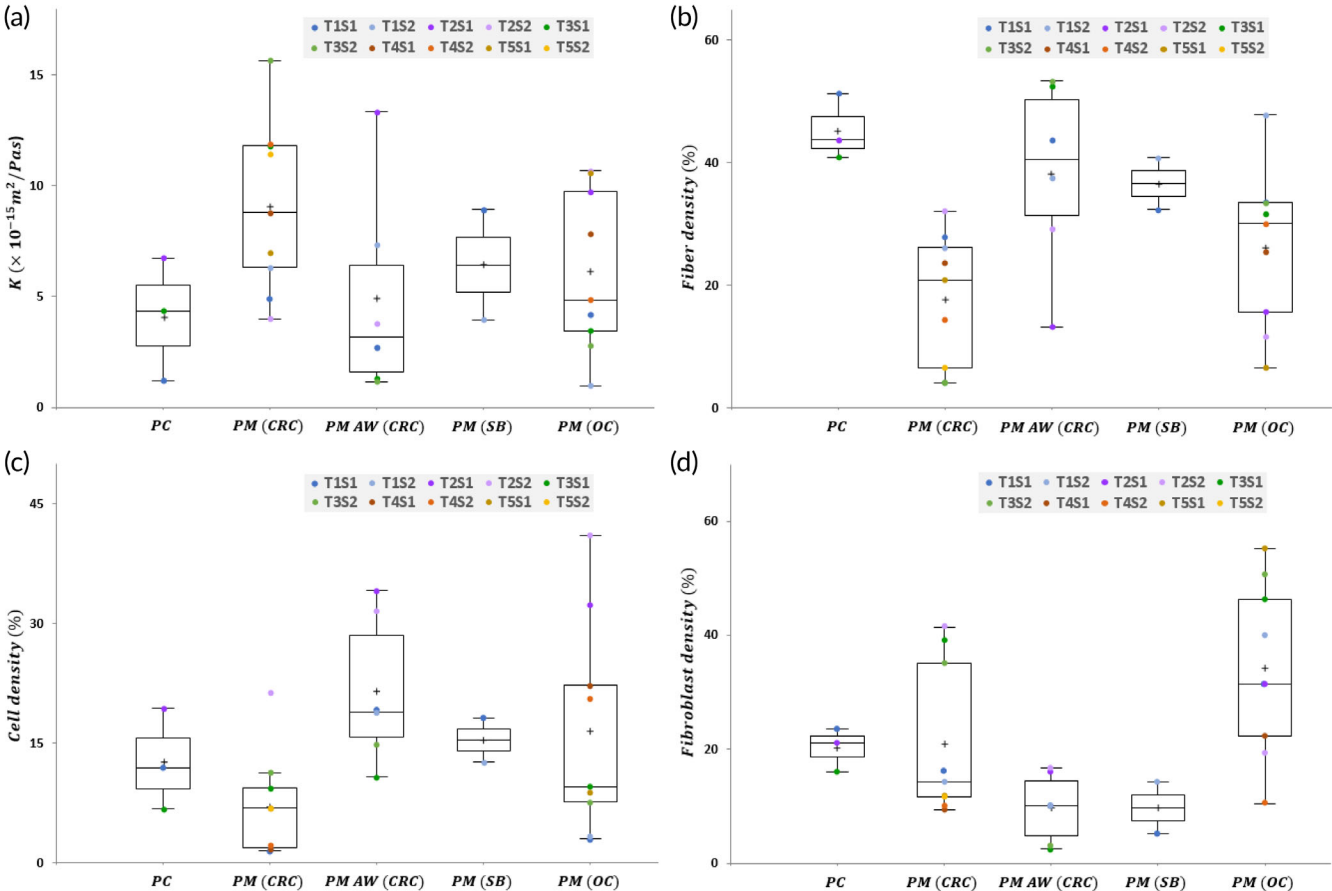
(a) Ex‐vivo results of the mean *K* values, (b) density of collagen fibers, (c) density of tumor cells, and (d) density of fibroblast per tumor type (Table 1), and individual values per sample (scatter plot; *N* = 29). T1S1 for instance stands for tumor No.1 and sample No.1, respectively. The “+” sign represents the mean value, and the box plots represent median values and the interquartile ranges.

A 3D box domain comprising a spherical solid tumor (with a diameter of Ø_t_ = 1 cm) with three distinct regions (necrotic, hypoxic, and viable) and surrounding normal tissue was considered for the numerical analysis (Figure 4a). The IFP was assumed to be normalized (i.e., IFP = 0 Pa) at the outer boundaries of the box (normal tissue). A constant drug concentration (*C* = 0.17 mol/m^3^) was prescribed to exist at the edge of the tumor regarding the typical range for cisplatin concentration in intraperitoneal chemotherapy.^61^ To investigate the effect of *K* heterogeneity in single tumors, a spatial profile (Figure 4b) was considered based on the work of Liu and Schlesinger,^48^ with *K* values as a function of location (Figure 4b), except in the necrotic core where a constant *K* value was used. In order to fit the suggested profile of *K* with the values from the ex vivo tests, three unknown variables (*K*_1_, *K*_2_ and *K*_normal_) were obtained from the ex vivo experiments. Since the S1 in each tumor was the most superficial slice, the values of *K*_1_ and *K*_2_ were equal to those measured for S1 and S2, respectively (Figure 5a).

The interstitial fluid flow and drug transport throughout the domain (Equations (1) and (10)) was simulated in COMSOL Multiphysics (COMSOL Inc., Burlington, USA) using a finite element method (FEM) solver. A parameter study on the values of *K* allowed investigating the impact of *K* on the interstitial fluid flow.

### 2.4 Statistical analysis

Simple linear regression models were fitted to the data to study correlations between the TME elements and between the TME elements and *K* values. The coefficient of determination (*R* ^2^) was calculated as a measure of model fit. Calculations and plotting were performed using Microsoft Excel™.

## 3 RESULTS

### 3.1 Experimental results

Measured *K* values (Figure 5; ranging between 9.5 × 10^−16^ and 1.6 × 10^−14^ m^2^/Pa∙s) demonstrated intra‐ and inter‐tumoral heterogeneity of *K* values. The variation of *K* within a single tumor ranged up to a factor of 4 in different locations, such as between PM (OC) T1S1 and T1S2. Also, significant variations of *K* were observed between different tumors of the same origin (up to one order of magnitude). The mean *K* in peritoneal metastases (PM) originating from colorectal or small bowel cancer was 7.3 ± 0.9 (SE) *×* 10^*−*15^ m^2^/Pa∙s, and the mean *K* value of PM from ovarian cancer was 6.1 ± 1.1 *×* 10^*−*15^ m^2^/Pa∙s. The values of *K* in the case of ovarian metastasis from small bowel cancer (6.3 ± 1.8 *×* 10^*−*14^ m^2^/Pa∙s) were in a different order of magnitude compared to the PM samples. This finding suggests the importance of the growth site in determining not only the biological properties (according to Paget's ‘seed and soil’ concept^62^), but also the biomechanical properties of cancer tissue. The results also indicated lower values of *K* in pancreatic cancer samples with a mean value of 4.1 ± 1.3 × 10^*−*15^ m^2^/Pa∙s. More importantly, the values of *K* of normal peritoneum (2.8 ± 0.7 × 10^*−*13^ m^2^/Pa∙s) were noticeably larger compared to peritoneal metastases.

The results of quantitative histology are illustrated in Figure 5. The highest density of collagen fibers was found in pancreatic cancer samples, with a mean value of 45.3 ± 2.5%, and subsequently in PM (CRC) infiltrated in the abdominal wall (AW) (38.3 ± 5.7%), followed by PM (OC) and PM (CRC) with mean values of 26.2 ± 4.1% and 17.8 ± 3.4%, respectively (Figure 5a). Significant variation was detected between fiber densities of PM samples, that is, up to 63% difference in samples from the same tumor and around 80% difference between different PM tumors. Comparing the values of cell density (Figure 5b), the mean value in PM infiltrated in the AW from CRC (21.5 ± 3.5%) was higher than the other tumor types followed by PM (OC), PC and PM (CRC), respectively.

The fibroblast density in the samples is illustrated in Figure 5c. The highest values were found in PM from OC (34.2 ± 4.7%) followed by PM from CRC (21.0 ± 4.2%). Interestingly, the fibroblast population in PM from CRC in the AW as well as in metastatic tumors from the small bowel (PM and OM) were noticeably smaller than the rest of PM samples. Generally the intra‐tumoral variation of fibroblast density was smaller compared to fiber and cell densities (Figure 5d).

When pooling all samples, no obvious correlation was found between the TME elements (i.e., collagen fibers, cells and fibroblasts). However, within separate tumor types, strong inverse correlations were present between the fiber and cell densities. An overview of observed correlations is listed in Table 3.

**TABLE 3:** The correlations observed between TME elements. Direct and inverse correlations were indicated with positive and negative values of parameter estimate (Par), respectively.

	Fiber (%) vs. cell (%)				Fiber (%) vs. fibroblast (%)				Cell (%) vs. fibroblast (%)			
	All, *N* = 29	PM (CRC), *N* = 9	PM AW (CRC), *N* = 6	PM (OC), *N* = 9	All, *N* = 29	PM (CRC), *N* = 9	PM AW (CRC), *N* = 6	PM (OC), *N* = 9	All, *N* = 29	PM (CRC), *N* = 9	PM AW (CRC), *N* = 6	PM (OC), *N* = 9
*r*	0.13	0.29	0.94	0.65	0.16	0.14	0.91	0.60	0.08	0.83	0.95	0.61
*p*	0.48	0.45	<0.001	0.06	0.40	0.71	0.01	0.09	0.69	<0.001	<0.001	0.08
SE	0.15	0.11	0.06	0.10	0.15	0.11	0.07	0.11	0.11	0.03	0.03	0.11
Int^a^	0.31	0.22	0.72	0.37	0.33	0.2	0.60	0.04	0.15	0.00	0.07	0.40
Par^b^	−0.19	−0.68	−1.54	−0.63	−0.19	−0.12	−2.27	0.69	−0.06	0.29	1.46	−0.72

^a^ Intercept.

^b^ Parameter estimate.

**FIGURE 6:**
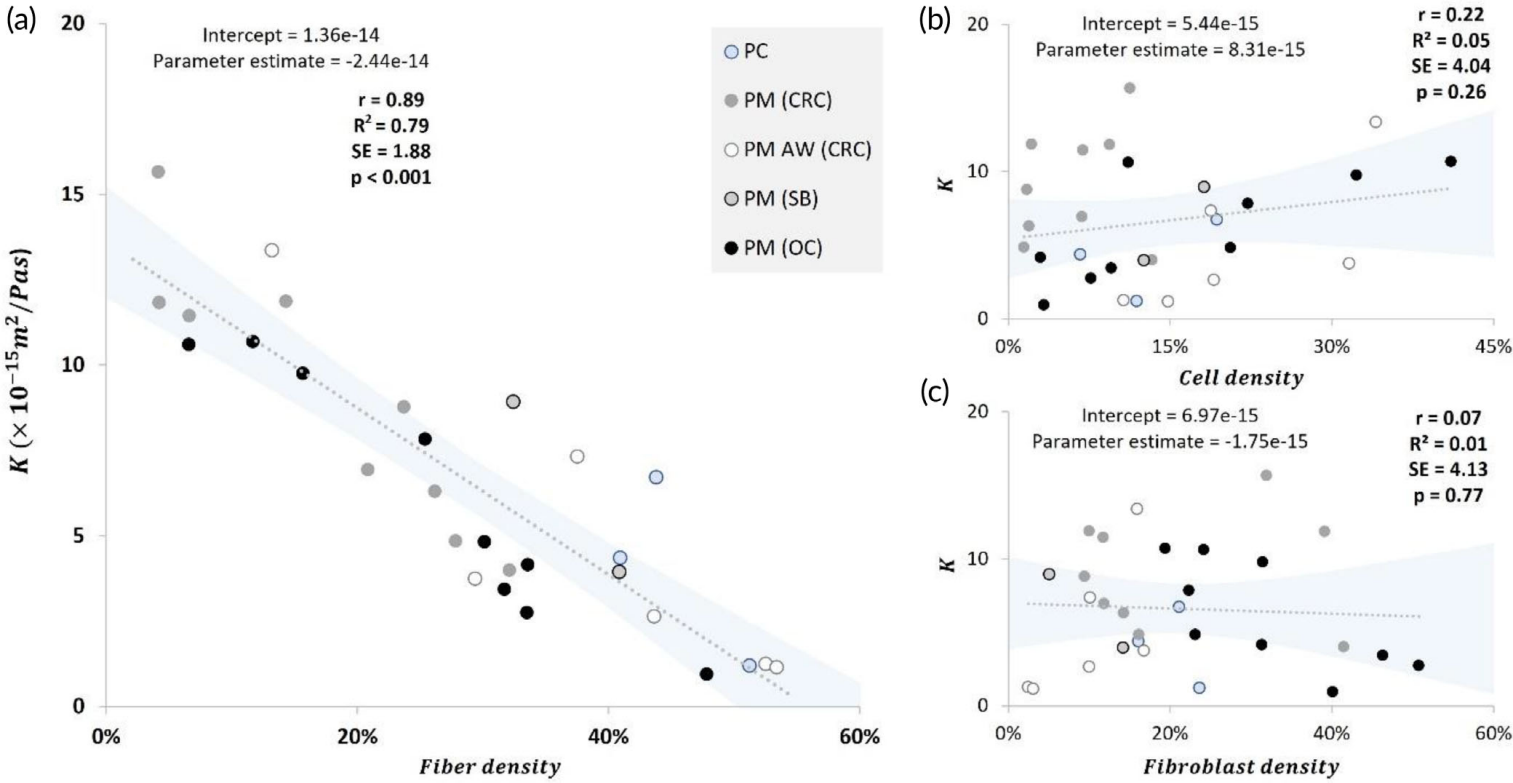
Correlation between the values of *K* and collagen fiber, cell and fibroblasts densities of samples (*N* = 29). The trendline of correlation and 95% confidence interval band were indicated by gray dotted line and blue shade, respectively.

Correlating the densities of TME elements with *K* values(Figure 6), a significant inverse correlation was found between collagen fiber density and the corresponding *K* values (*R* ^2^ = 0.79, *p* < 0.0001) when grouping the data for all tumor types (Figure 6a). Focusing on the origin and growth site of the tumor, stronger correlations were also observed for instance in PM from CRC (*R* ^2^ = 0.86, *p* < 0.0001) or OC (*R* ^2^ = 0.93, *p* < 0.0001). Analyzing the cell density relation with *K* (Figure 6b), no general relation was found to exist between the tumor cell density and *K*. However, several moderate direct correlations were detected among cell density and *K* within defined tumor types, for instance in PM (CRC) and PM (OC). Similarly, no correlation was found to exist between the fibroblasts content and *K* (Figure 6c; *R* ^2^ = 0.01, *p* = 0.77). Nevertheless, weak inverse correlations were found between fibroblast density and *K* values in samples of PM from CRC and OC.

### 3.2 CFD modeling

**FIGURE 7:**
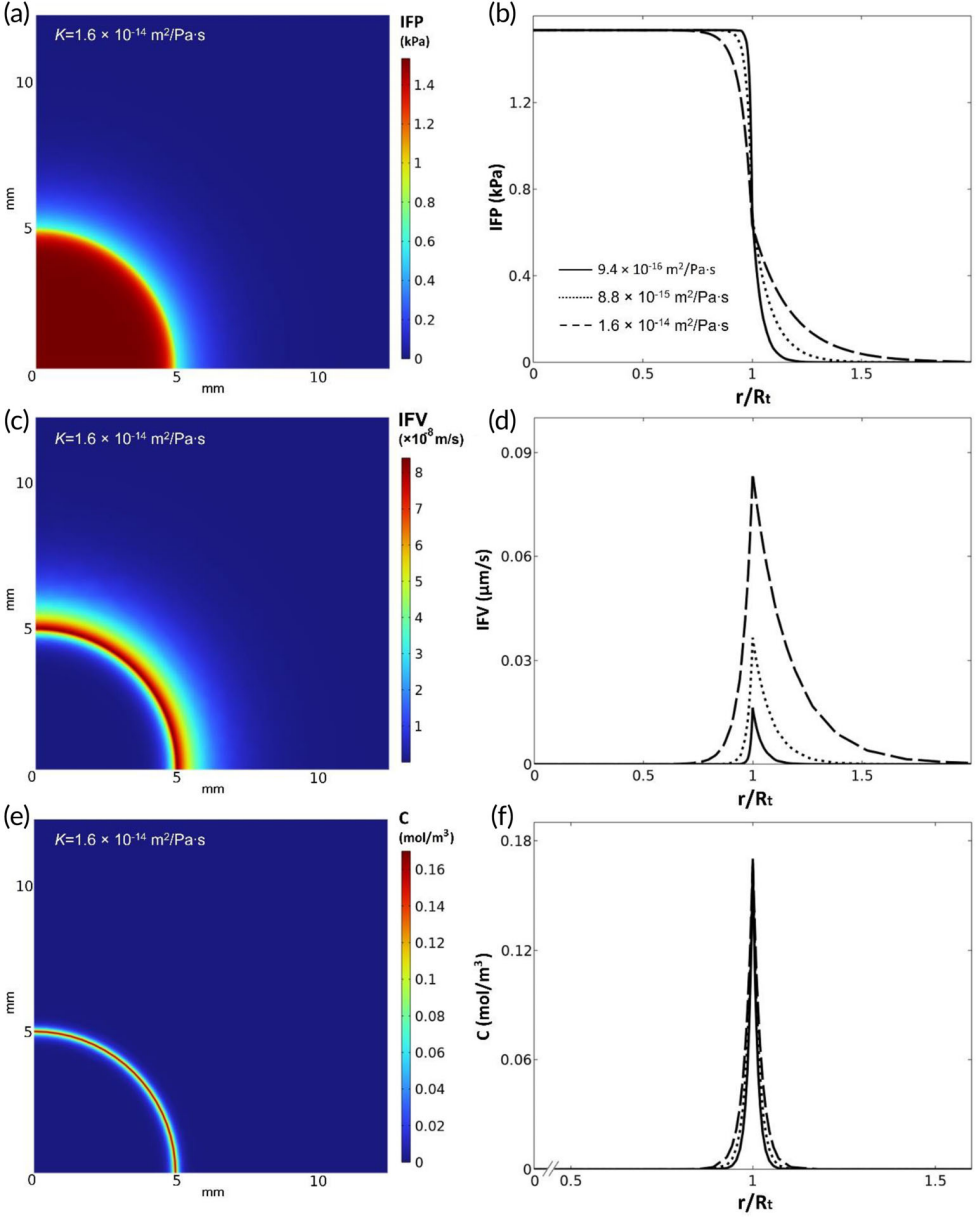
(a) IFP, (c) IFV and (e) drug concentration distributions for *K* = 1.6 × 10^−14^ m^2^/Pa∙s, and the effects of *K* variations on (b) IFP, (d) IFV and (f) drug concentration.

For the CFD model, a parameter study was performed regarding the observed heterogeneity in *K* values between different tumors (the growth site or tumor type) (Figure 7). The variation of *K* values in different tumor types and the resulting effect of tumor interstitial fluid flow (IFP and IFV) and drug penetration were simulated using three nominal *K* values, i.e., PM (OC) T1S2 (9.4 × 10^*−*16^ m^2^/Pa∙s), PM (SB) T1S1 (8.8 × 10^*−*15^ m^2^/Pa∙s) and PM (CRC) T3S2 (1.6 × 10^*−*14^ m^2^/Pa∙s) that were obtained from the ex‐vivo experiments. In all cases, to ensure a smooth transition from cancerous to normal properties, the value of *K* in normal tissue (*K*_normal_) was considered to be five times greater than the value of *K* inside tumor.

The 2D plots of IFP, IFV and drug concentration for PM (CRC) T3S2 are shown in Figure 7a,c,e, respectively. Figure 7b illustrates the impact of *K* on the IFP inside solid tumors. The highest IFP was similar in all three cases (1.5 kPa), but significant differences were detected in the IFP profile, specifically close to the edge of the tumor (*r*/*R*_t_ = 1), where a lower *K* corresponded with a higher IFP value. A turning point was detected at the vicinity of the tumor edge, where the trend of IFP regarding *K* was inversed in the normal tissue surrounding the tumor, having the higher value of IFP with the highest *K*. Interestingly, the values of IFV (Figure 7d) were generally higher with higher values of *K* due to the larger IFP gradient. Furthermore, the extent of non‐zero IFV values increased with higher *K* values. The drug concentration results indicated a limited penetration depth (Figure 7e), while illustrating a deeper penetration depth with higher *K* values (Figure 7f). For instance, the non‐zero concentration zone for the highest *K* (Figure 7f; dashed line) was approximately twice as large as that for the lowest *K* (Figure 7f; solid line).

**FIGURE 8:**
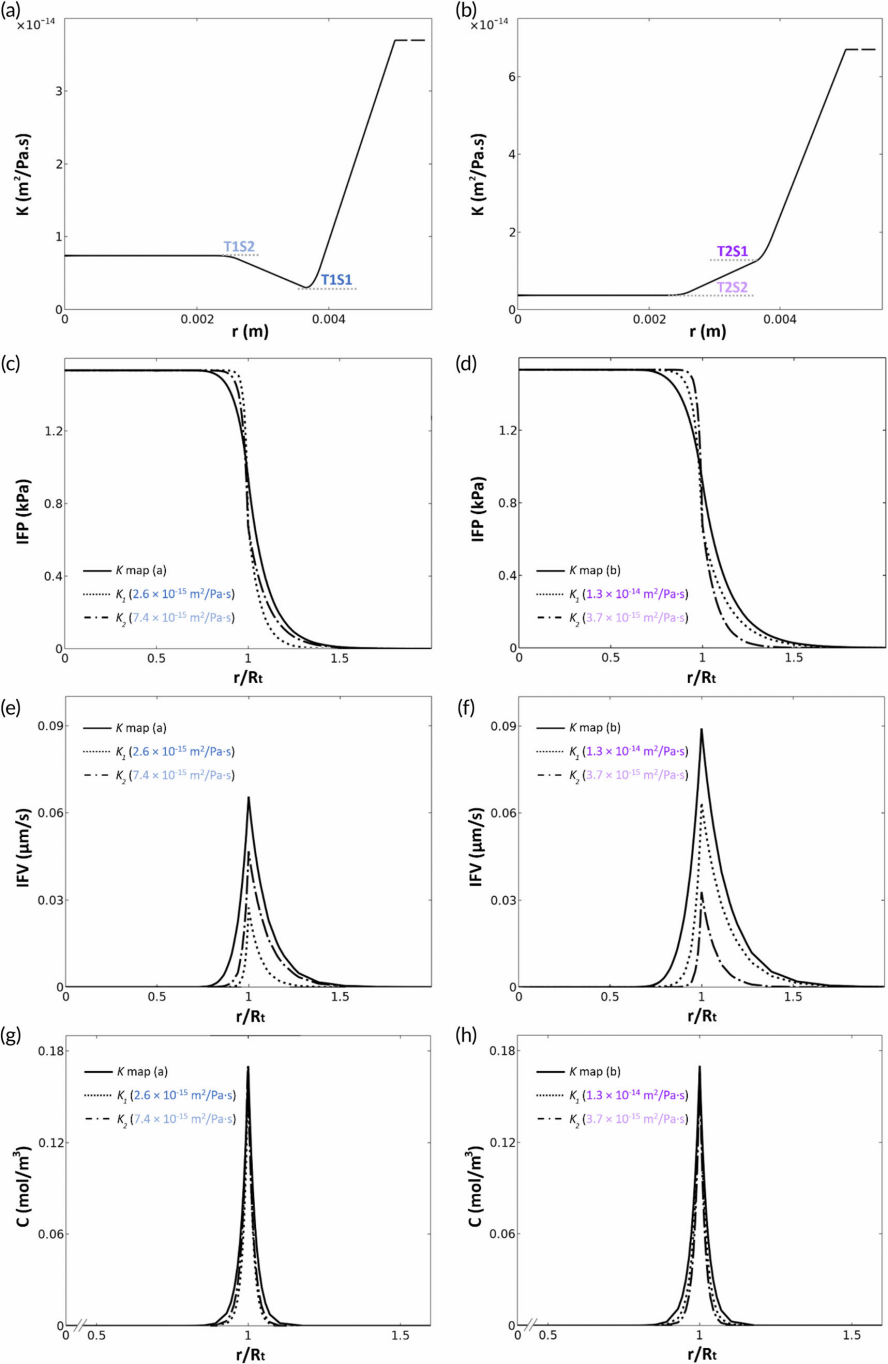
(a,b) The prescribed *K* maps based on ex‐vivo results of two PMs (CRC) infiltrated in AW. (c,d) IFP, IFV (e,f) and (g,h) drug concentration profiles using *K* maps (solid lines) and homogenous *K* values (dotted and dash‐dot lines).

As shown in Figure 5, *K* can vary spatially within single tumors. To study the impact of this heterogeneity on tumor perfusion and drug penetration, the values of *K*_1_ and *K*_2_ in the profile depicted in Figure 4b were determined based on experimental values of *K* in two PMs (CRC) infiltrated in the AW (T1 and T2), resulting in two *K* profiles Figure 8a,b, respectively. In both cases, the IFP profile (Figure 7c,d) assuming a heterogeneous *K* profile (solid line) is generally lower inside the tumor and higher in normal tissue compared to assigning homogeneous values. Additionally, the transition of IFP from high values in the central region of the tumor to normal IFP was smoother with the heterogeneous *K* profiles, decreasing the plateau IFP region. Additionally, the IFV profile with a heterogeneous *K* is generally higher than in the homogeneous cases. The drug penetration depth was slightly enhanced when using the heterogeneous *K* maps. Comparing the results of the two *K* maps (Figure 8a,b), the IFP, IFV, and drug concentration were more variable in T2 compared to T1.

## 4 DISCUSSION

There is increasing interest in the mechanobiology of cancer.^63^ The structural properties of the tumor associated extracellular matrix have a major impact on cancer initiation, progression, and metastasis. Also, drug delivery is known to be hampered by elevated interstitial fluid pressure and excessive deposition of matrix fibers. Advances in functional imaging have opened the prospect of estimating the spatial distribution of tumor tissue IFP. The functional information thus provided could represent a novel biomarker guiding therapy directed against the tumor associated stroma.^64^

Non‐invasive measurement of the IFP requires knowledge of the tissue hydraulic conductivity, which is a property of porous materials and depends on the intrinsic permeability of the tissue and on the density and viscosity of the intercellular fluid. Thus far, values of *K* were derived from animal studies or estimated indirectly. The current study is the first to measure *K* in human tumor samples from various origins. Several key findings can be mentioned. First, the hydraulic conductivity of tumor tissue is very low (5–10 x 10^−15^ m^2^/Pa·s), and comparable to the *K* of dense rock. This property of cancer tissue contributes to the limited penetration depth of anticancer drugs, which is typically 30–40 μm, leaving significant portions of the tumor cell population untreated.^65^, ^66^ The values for *K* that we observed in human samples were generally lower compared to published estimates from animal models and computational models.^30^, ^39^, ^40^, ^41^, ^42^, ^67^, ^68^, ^69^, ^70^ This difference can be explained by dissimilarities in tumor tissue architecture between rodent xenografts and human cancer, and using buffers with a lower viscosity than interstitial fluid. In the published literature, *K* is variously reported as either lower or higher compared to normal tissue.^31^, ^48^, ^71^, ^72^ Using human samples, we found that values of *K* in normal tissue are one order of magnitude higher compared to tumor tissue. These experimental observations were corroborated by the CFD model (Figure 8a,b).

Second, hydraulic conductivity varies considerably within and between tumor type and location: in a single tumor type, *K* differed between samples from 11% to 72%. Therefore, for the purpose of mathematical modeling, using a single fixed value for *K* will likely lead to imprecise estimates of tumor interstitial pressure and drug delivery.

Third, we found a significant inverse correlation between *K* and the collagen fiber content in all tested samples. Besides hindering interstitial fluid flow, dense collagen fibers also limit mechanical deformation, further affecting the hydraulic conductivity.^73^ Previous animal studies have demonstrated that structural elements including collagen and glycosaminoglycans are the main tissue components regulating tissue deformation and resistance against fluid flow.^25^, ^69^ In human pancreas cancer, the adverse effect of the desmoplastic stroma on drug delivery is well established and has stimulated the development of stromal targeting therapies.^74^

We did not find a correlation between *K* and the cancer cell density. However, this finding should be interpreted with caution given the paucicellular nature of the tested samples, and the difficulty of accurately outlining cancer cells on histology slides.

The results of the CFD model demonstrated the controlling effect of *K* on IFP as well as on IFV inside tumor tissue, both known as mechanical biomarkers of cancer. Moreover, the model showed that *K* has a significant impact on simulated penetration depth of chemotherapy, as it is assumed to determine the effective diffusivity of the drug within the tissue. The results showed that variation of *K* among different tumor types significantly affects the IFV profile, which is the driving force of convective drug transport. Furthermore, simulating *K* as a heterogeneous property in tumors was shown to result in different profiles of IFP, IFV, and drug concentration. The current study can provide important information for future computational models of tumor perfusion and drug delivery. Most computational studies of interstitial fluid flow and drug delivery to solid tumors have used a single value of *K*, regardless of the tumor type or anatomical location.^22^, ^75^, ^76^, ^77^, ^78^, ^79^, ^80^ To our knowledge, the current work is the first to prescribe a profile for *K* based on experimental results. A considerable variation in the IFP profiles obtained from the heterogeneous *K* is noted when compared to the common assumption in the literature assigning a single value of *K* to the whole tumor.

Some limitations apply to the interpretation of this work. First, although a sizeable number of samples was tested, the observed heterogeneity suggests that a larger sample size will probably allow to draw more robust conclusions on the average hydraulic conductivity of separate tumor types. Also, samples were measured ex vivo, obviously implying that the tissue is devoid of a vascular blood supply during the biomechanical measurements, and this may introduce a bias. While direct validation of the simulation results against experimental observations is not straightforward due to the difficulties in clinically measuring IFP, IFV, and drug penetration, comparisons with previous observations on IFP and IFV values^6^, ^49^, ^79^, ^81^, ^82^ demonstrate a close similarity with the current study, affirming the validity of the computational model.

In conclusion, hydraulic conductivity of human cancer tissue is heterogeneous and very limited, and contributes to elevated IFP and possibly to poor drug penetration. Conductivity is inversely related with the collagen fiber content, highlighting the potential of stromal targeting therapies in desmoplastic tumors. The present results may facilitate the development of such therapies, and inform methods for noninvasive estimation of tumor tissue biomechanical parameters including interstitial fluid pressure and velocity.

## AUTHOR CONTRIBUTIONS

**Hooman Salavati:** Conceptualization (supporting); formal analysis (lead); investigation (lead); methodology (supporting); software (lead); visualization (lead); writing – original draft (lead); writing – review and editing (supporting). **Pim Pullens:** Investigation (supporting); supervision (supporting); writing – review and editing (supporting). **Charlotte Debbaut:** Investigation (supporting); software (supporting); supervision (equal); visualization (supporting); writing – review and editing (supporting). **Wim Ceelen:** Conceptualization (lead); investigation (supporting); methodology (lead); supervision (lead); visualization (supporting); writing – review and editing (lead).

## FUNDING INFORMATION

This work was funded by grants from Stichting tegen Kanker (project 2018‐104) and the Research Foundation—Flanders (FWO, project number 3G020919).

## CONFLICT OF INTEREST STATEMENT

The authors declare that they have no competing interests relating to this work.

### PEER REVIEW

The peer review history for this article is available at https://www.webofscience.com/api/gateway/wos/peer-review/10.1002/btm2.10617.

## Supporting information


**Appendix S1:** Supporting information.

**TABLE S1.** Parameters values used for CFD simulation.

## Data Availability

The data that support the findings of this study are available from the corresponding author upon reasonable request.
