# Supplementary material for: Hydraulic conductivity of human cancer tissue: A hybrid study
Source: Bioeng Transl Med. 2023 Nov 23;9(2):e10617. doi: 10.1002/btm2.10617 (PMC10905546; doi:10.1002/btm2.10617)
Supplement: Supplementary file 1 — Appendix S1: Supporting information. [file BTM2-9-e10617-s001.docx]

**Supplementary materials**

Staining techniques

To quantify the cell density, samples were hematoxylin and eosin (H&E) stained. Briefly, after 5 minutes of deparaffinization in xylene, the slices were run through 99% ethanol alcohol (EtOH) and 96% EtOH for 10 minutes each, and distilled water (dH_2_O) for 5 minutes, respectively. Afterwards, the slices were placed in hematoxylin for 5 minutes, followed by rinsing for 6 minutes with water. Samples were then placed in 96% EtOH for 1 minute, and 1% eosin was applied on for 5 minutes.

Masson’s trichrome stains all types of collagen, as well as cell cytoplasms and cell nuclei. The slices were first placed in Bouin’s kept at 56C for 15 minutes, and then rinsed under running tap water. The sections were stained with the iron hematoxylin for 5 minutes afterwards followed by 2 minutes rinsing under water. Next the slices were stained with Biebrich scarlet-acid fuchsin for 5 minutes. After the sections were further rinsed in deionized water, they were treated with phosphomolybdic solution for 5 minutes. The sample then were run through Aniline blue for 5 minutes and Acetic acid solution for 2 minutes, respectively.

Alpha-smooth muscle actin (ASMA) stains also detects the fibroblasts in TME. To perform ASMA stain, antigen retrieval was performed by Citrate buffer (pH 6.0), and consequently sample were treated in Wavy-walled bioreactor (WWB) at 95C for 20 minutes. Sections were cooled down and rinsed with PBS followed by blocking of nonspecific binding with 5% normal rabbit serum for 30 minutes. The slides then treated with immunostaining with anti-alpha smooth muscle actin diluted 1:800 overnight. Sections were rinsed in PBS, incubated with biotinylated rabbit anti-mouse IgG (peroxidase) polymer detection reagent for 10 minutes and a solution of the Streptavidin Peroxidase diluted 1:200 in PBS with 0.05% Tween 20. The slices afterwards were stained with DAB + chromogen solution for 10 minutes. Sections were counterstained with hematoxylin followed by water rinsing.

In H&E stain, cell nuclei are stained purple, and cytoplasm and extracellular matrix have varying shades of pink. In Masson’s trichrome stain, collagen fibers were stained green and the blue and red stains were nuclei and cytoplasm, muscle or erythrocytes, respectively. For the ASMA stain, fibroblasts expression were detected by the brown stain.
