# Supplementary material for: Hydraulic conductivity of human cancer tissue: A hybrid study
Source: Bioeng Transl Med. 2023 Nov 23;9(2):e10617. doi: 10.1002/btm2.10617 (PMC10905546; doi:10.1002/btm2.10617)
Supplement: Supplementary file 2 — TABLE S1. Parameters values used for CFD simulation. [file BTM2-9-e10617-s002.docx]

**Supplementary Table 1.** Parameters values used for CFD simulation

| **Parameter** |  | **Baseline value** | **Reference** |
| --- | --- | --- | --- |
| $L_{p}$ $[m/Pa s]$ | Tumor | $2.1\times{10}^{-11}$ | [7] |
|  | Normal | $2.7\times{10}^{-12}$ |  |
| $\frac{S}{V}$ $[m^{-1}]$ | Tumor | $7.0\times{10}^{3}$ | [7] |
|  | Normal | $2.0\times{10}^{4}$ |  |
| $p_{v}$ $[Pa]$ | Tumor & Normal | $2080$ | [7] |
| $\pi_{v}$ $[Pa]$ | Tumor & Normal | $2666$ | [7] |
| $\pi_{i}$ $[Pa]$ | Tumor | $2000$ | [7] |
|  | Normal | $1333$ |  |
| $\sigma$ | Tumor | $0.8$ | [7] |
|  | Normal | $0.9$ |  |
| $L_{p_{L}}\frac{S_{L}}{V_{L}}$ $[1/Pa s]$ | Tumor | 0.0 | - |
|  | Normal | $1.0\times{10}^{-7}$ | [52] |
| $p_{L}$ $[Pa]$ | Tumor & Normal | 0.0 | [52] |
| $D_{eff}^{^{\circ}}$ $[m^{2}/s]$ | Tumor & Normal | $2.5\times{10}^{-10}$ | [45] |
| $K^{^{\circ}}$ $[m^{2}/s]$ | Tumor & Normal | $8.9\times{10}^{-15}$ | [45] |
| $L_{d}$ $[m/s]$ | Tumor | $1.7\times{10}^{-8}$ | [7] |
|  | Normal | $2.2\times{10}^{-9}$ |  |
| $\sigma_{D}$ | Tumor & Normal | 0.9 | [7] |
